# The Assessment of Autonomic Nervous System Activity Based on Photoplethysmography in Healthy Young Men

**DOI:** 10.3389/fphys.2021.733264

**Published:** 2021-09-24

**Authors:** Binbin Liu, Zhe Zhang, Xiaohui Di, Xiaoni Wang, Lin Xie, Wenjun Xie, Jianbao Zhang

**Affiliations:** Key Laboratory of Biomedical Information Engineering of Ministry of Education, Institute of Health and Rehabilitation Science, School of Life Science and Technology, Xi'an Jiaotong University, Xi'an, China

**Keywords:** photoplethysmography, autonomic nervous system, cardiovascular system, heart rate variability, classification, noninvasive assessment technique

## Abstract

Noninvasive assessment of autonomic nervous system (ANS) activity is of great importance, but the accuracy of the method used, which is primarily based on electrocardiogram-derived heart rate variability (HRV), has long been suspected. We investigated the feasibility of photoplethysmography (PPG) in ANS evaluation. Data of 32 healthy young men under four different ANS activation patterns were recorded: baseline, slow deep breathing (parasympathetic activation), cold pressor test (peripheral sympathetic activation), and mental arithmetic test (cardiac sympathetic activation). We extracted 110 PPG-based features to construct classification models for the four ANS activation patterns. Using interpretable models based on random forest, the main PPG features related to ANS activation were obtained. Results showed that pulse rate variability (PRV) exhibited similar changes to HRV across the different experiments. The four ANS patterns could be better classified using more PPG-based features compared with using HRV or PRV features, for which the classification accuracies were 0.80, 0.56, and 0.57, respectively. Sensitive features of parasympathetic activation included features of nonlinear (sample entropy), frequency, and time domains of PRV. Sensitive features of sympathetic activation were features of the amplitude and frequency domain of PRV of the PPG derivatives. Subsequently, these sensitive PPG-based features were used to fit the improved HRV parameters. The fitting results were acceptable (*p* < 0.01), which might provide a better method of evaluating ANS activity using PPG.

## Introduction

The autonomic nervous system (ANS) plays a crucial role in the regulation of human function. Excessive sympathetic activation is associated with numerous cardiovascular diseases and even sudden death (Lahiri et al., 2008). Therefore, the assessment of ANS activation is of great significance. Electrocardiogram (ECG)-derived heart rate variability (HRV) is used widely for the evaluation of ANS activation. In addition, photoplethysmography (PPG) also offers a wealth of cardiovascular information (Allen, 2007). Therefore, PPG-derived pulse rate variability (PRV) is considered as an alternative measure to HRV. Related research has included data from different experimental states, such as body positions (supine, tilting, and standing) (Charlot et al., 2009; Gil et al., 2010; Iozzia et al., 2016), whole-body cold exposure (Mejía-Mejía et al., 2020), and mental stress (Giardino et al., 2002; Pernice et al., 2019). A common conclusion is that PRV and HRV are interchangeable at resting state. However, their substitutability is distinctly reduced or even destroyed during most task states. Furthermore, another issue that is often overlooked is the inaccuracy of HRV itself, especially sympathetic-related parameters (Billman, 2013), which may further limit the application of PRV in estimating ANS activity.

In addition to PRV, PPG offers further information, which includes blood pressure (Liang et al., 2019), systemic vascular resistance (Awad et al., 2007; Wang et al., 2009), arterial tone and stiffness (Allen and Murray, 2003; Millasseau et al., 2006), and vasomotor responsiveness (McVeigh et al., 1997). Moreover, its first (velocity of PPG, VPG) and second derivatives (acceleration of PPG, APG) are closely related to vascular status (Takada et al., 1996; Imanaga et al., 1998; Takazawa et al., 1998), which have been applied to examinations of aging (Bortolotto et al., 2000; Baek et al., 2007), carotid distensibility (Imanaga et al., 1998), arterial stiffness in adolescents (Miyai et al., 2001), and treated hypertensive patients (Hashimoto et al., 2002). It is believed that the derivatives of PPG are more sensitive to small changes in PPG (Elgendi, 2012); however, only PPG features are considered when identifying specific physiological and pathological states, such as low-stress states (Pelaez et al., 2019) and postoperative pain (Seok et al., 2019). Since ANS is the main regulatory system of the cardiovascular system, ANS assessment may be more accurate if features of PPG and its derivatives are combined.

Machine learning is a promising method for comprehensively utilizing multiple types of information and has been applied to other physiological signals, such as electroencephalogram and ECG (Hu et al., 1997; Wang et al., 2014). However, most classification models cannot be used to determine underlying mechanisms because of their “black box” characteristics. Breiman (2001) proposed an efficient ensemble algorithm named random forest (RF), whose internal estimation can measure the feature importance. Based on the RF model, Genuer et al. (2010) designed an interpretable model that guarantees good classification performance and obtains the most sensitive features for physiological states associated with the classification.

The purpose of this study was to explore the feasibility of using PPG for ANS evaluation. We implemented the classification of four ANS patterns using 110 PPG-based features by innovatively introducing the features of PPG derivatives. Based on the interpretable RF model, the most sensitive features for sympathetic and parasympathetic activity were identified. We then developed a regression model with these sensitive features to estimate the improved HRV parameters to provide a better method of evaluating ANS activity using PPG.

## Materials and Methods

### Data Acquisition

We recruited 32 healthy, young, male volunteers aged between 19 and 26 years (23.2 ± 2.9 years). Participants abstained from smoking, consuming caffeine and alcohol, and performing heavy physical exercise for 24 h before the test. The study was conducted in accordance with the Helsinki Declaration of 1975 (as revised in 2008) concerning human and animal rights and was approved by the ethics committee of the Xi'an Jiaotong University. Written informed consent was obtained from all subjects.

Each subject participated in four experiments (E1 to E4) corresponding to the four different patterns of ANS activation in the body.

E1 was baseline (BSL), which referred to a resting state with no particular branch of ANS being activated, and subjects sat quietly while keeping their eyes open.

E2 involved slow deep breathing (SDB), whereby the parasympathetic branch of the ANS was activated; this has been used as a stress-reduction technique previously (Gilbert, 2003). Subjects closed their eyes while in a sitting position. To avoid mental concentration induced by controlled breathing (McClain et al., 2017), no specific respiratory rate was set. Subjects were encouraged to slow down and deepen their breathing as long as they felt relaxed and comfortable. Data acquisition was performed after 10 min of respiratory training.

Mental arithmetic (MAT) in E3 referred to a state where the cardiac sympathetic branch of ANS was activated (Wang et al., 2016). Participants were seated in front of a monitor and performed continuous subtraction operations using the minuend and subtrahend presented on the monitor. The minuend was a random integer between 800 and 900, and the subtrahend was 7, 9, and 13 for the three rounds, respectively, which changed every 100 s to prevent subjects from getting used to the operation and decreasing their attention.

The cold pressor test (CPT) in E4 referred to a state where the peripheral sympathetic branch of ANS was activated (Silverthorn and Michael, 2013). Subjects were asked to immerse their right hand (the part below the wrist) into cold water with a temperature of 4–5°C while in a sitting position.

The acquisition duration of each experiment was 5 min, without physical movement or talking, followed by a 15-min rest before the next experiment, during which time subjects were permitted to stand if they felt uncomfortable. All experiments were conducted in a silent and temperature-controlled room (25°C) between 2 and 4 p.m.

Photoplethysmography (index finger of the left hand), one-lead ECG, and chest band-based respiratory signals were recorded at a sampling rate of 1,000 Hz (MP150; BIOPAC Systems, Goleta, CA, USA). The measurement module of PPG was OXY100E, which was operated in accordance with the principles outlined in the Lambert–Beer law. The OXY100E probe incorporates light-emitting diodes (LEDs), which include one red LED with a wavelength of 660 nm and one infrared LED with a wavelength of approximately 910 nm. Electrode connections were made according to the MP Hardware Guide. Because of the aberrant PPG waveform in two SDB and four CPT recordings, which made it impossible to extract feature points, data from four subjects were excluded from the following analyses.

### Signal Preprocessing and Feature Extraction

All computations in this section were executed using MATLAB R2015b.

#### ECG Signal Preprocessing, Traditional HRV, and Improved HRV Calculation

The wavelet transform was used to remove baseline drift (below 0.122 Hz) and high-frequency noise (over 31.25 Hz) from the ECG signal. Then, R peaks were detected using the wavelet modulus maximum algorithm (Li et al., 1995). To avoid false detection, artifact modification and rejection were performed manually. Continuous R–R interval (RR) sequences were converted into heart rate (HR) using 60/RR. HR was then resampled at 4 Hz and divided by its average value before frequency analysis to remove the mathematic impact of mean HR (Sacha, 2013).

Subsequently, Morlet-wavelet transform (MWT) with the mother wavelet “cmor3-1” (Wang et al., 2016) was implemented on the processed HR to obtain the frequency-dependent complex analytic signal *y*(*t, f*), and the time-frequency power spectrum of which was calculated as Equation 2.1 (Schiecke et al., 2014):


(2.1) $$\begin{array}{r} ps\left(t,f\right)=\;|y\left(t,f\right){|}^{2} \end{array}$$

Then, the traditional HRV parameters, low-frequency power (LF, 0.03–0.15 Hz), high-frequency power (HF, 0.15–0.4 Hz), and total power (TP) of HRV were acquired by integrating *ps*(*t,f*) within their corresponding frequency range. Normalized LF [nLF = LF/(LF+HF)] and LF/HF were also calculated. It is generally believed that HF represents parasympathetic activity, nLF represents sympathetic activity, and LF/HF represents sympathovagal balance (Berntson et al., 1997).

Because traditional HRV does not always correctly represent ANS activity, an improved HRV method based on time-variant cardiorespiratory relation was proposed (Liu et al., 2019). Taking respiratory signal as a reference, the original HR is divided into a respiratory-related HR component (HRr) and a respiratory-unrelated HR component (HRru), for which the spectrum transforms are HFr and LFru, respectively. Further calculations of nLFru [*nLFru* = *LFru/*(*LFru* + *HFr*)] and LFru/HFr were also performed. HFr represents parasympathetic activity, nLFru represents sympathetic activity, and LFru/HFr represents sympathovagal balance.

#### PPG Signal Preprocessing and Feature Calculation

**Figure 1:**
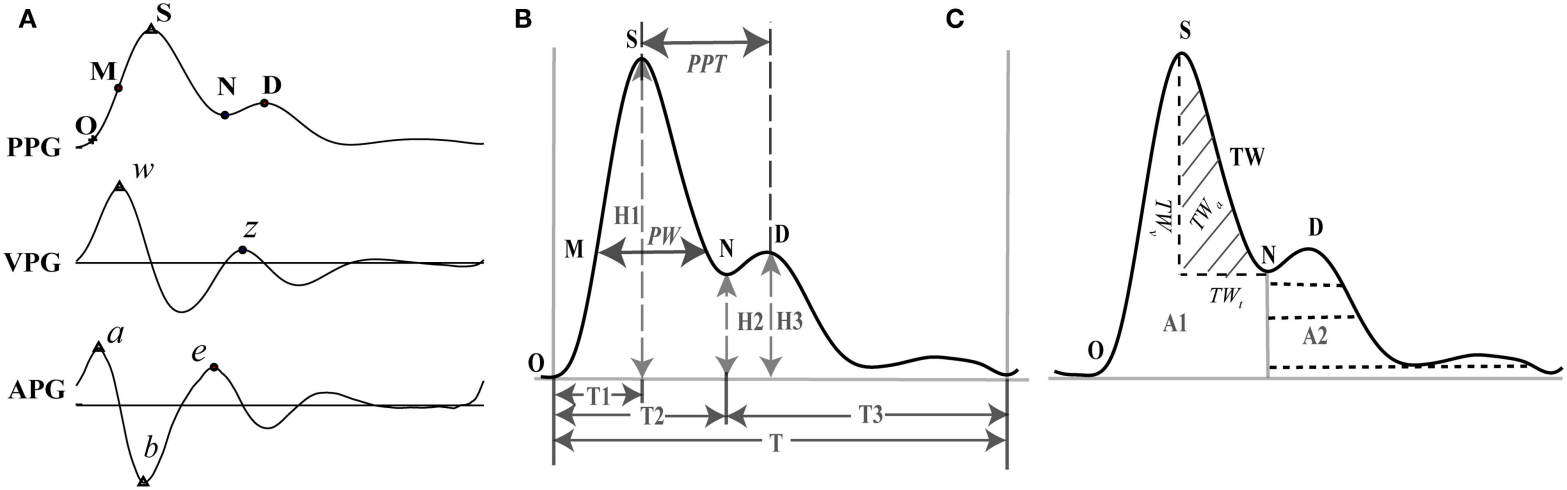
Feature extraction of PPG and its derivatives. **(A)** Feature points on PPG, VPG (first derivative or velocity of PPG), and APG (second derivative or acceleration of PPG). **(B)** Representative features of the PPG. **(C)** Representative features of the PPG. O: onset of the systolic wave; S: the systolic peak; M: the midpoint of the systolic peak; N: the dicrotic notch; D: the diastolic peak; w: the maximum slope point in systole; z: the maximum slope point in diastole. a: the maximum acceleration point in systole; b: the maximum negative acceleration point in the falling edge; e: the maximum acceleration point in diastole. PW, pulse width corresponding to the midpoint of the rising phase; PPT, peak to peak interval between the systolic and diastolic peaks; TW, tidal wave; *TW*_*t*_, time range of TW; *TW*_*v*_, amplitude of TW; *TW*_*a*_, area of TW.

Frequency components of the PPG signal beyond 0.122–15.625 Hz were removed using wavelet transform. The first and second derivatives of PPG (VPG and APG) were calculated. The characteristic points in the waveforms were named according to Elgendi et al. (2018) whereby 10 typical points were selected that could be accurately discerned from the PPG, VPG, and APG waveforms (Figure 1A).

One heartbeat PPG waveform can be divided into two phases: the rising edge primarily concerned with systole and the falling edge with diastole and wave reflections from the periphery. Five points were extracted from the PPG waveform: onset of the systolic wave (O), the systolic peak (S), the midpoint of the systolic peak (M), the dicrotic notch (N), and the diastolic peak (D) (Elgendi, 2012). For the VPG waveform, the two most prominent points were identified: the maximum slope point in systole (w) and the maximum slope point in diastole (z). Point w is recommended as the most appropriate reference point for calculating HR (Suhrbier et al., 2006; Peralta et al., 2019), and point z has great auxiliary value for the extraction of points N and D from the PPG waveform. Each heartbeat of the APG waveform also consists of five characteristic points (a, b, c, d, and e), all of which are sensitive to vascular status (Takazawa et al., 1998). However, in practice, points c and d are difficult to extract accurately. To ensure repeatability of the study, we extracted only points a, b, and e.

According to the above definition, points S, w, a, and b were identified by searching for the extreme points on the corresponding signals within 400 ms of the R peak of the ECG. Then, points e and z were detected by finding the maximum point within 200 ms of point b. For detecting points N and D, two strategies were used (Millasseau et al., 2002, 2003). If the value of z was > 0, point N was positioned at the zero-crossing point before z, and point D was positioned at the zero-crossing point after z. Otherwise, point D was positioned at point z, and point N was positioned in the middle between points e and z. Point O was identified using the intersecting technique (Chiu et al., 1991). First, the point of the maximum first derivative on the PPG was identified. Points were then added on both sides of this point until the correlation coefficient between the actual signal and the fitted line became <0.999. Point O was determined by the intersection of this line and a horizontal line that passed through the minimum point of the corresponding PPG segment. To avoid false detection, visual inspection and manual modification were carried out after automatic detection.

After feature point extraction, a total of 110 PPG-based features were obtained, which consisted of 80 PPG features, 24 VPG&APG features, and 6 pulse rate (PR)-related features (Table 1).

**Table 1:** The 110 features extracted from PPG, VPG (first derivative or velocity of PPG), APG (second derivative or acceleration of PPG), and PRV (pulse rate variability).

**PPG**	**Interval**	**1: T (time span of the pulse)**	**6: PPT (interval between the systolic [S] and diastolic peaks [D])**
		**2: T1 (rising time of the pulse)**	**7: T1/T**
		**3: T2 (systolic time of the pulse)**	**8: T2/T (the same as T3/T)**
		**4: T3 (diastolic time of the pulse)**	**9: PW/T**
		**5: PW (pulse width corresponding to the midpoint of the rising phase)**	**10: PPT/T**
	**Area**	**11: A1 (area of systolic phase)**	**14: IPA: A2/A1 (diastolic area/systolic area)**
		**12: A2 (area of diastolic phase)**	**15: A1/A**
		**13: A (A1 + A2, area of the pulse)**	
	**Height**	**16: H1 (height of the systolic peak)**	**19: H2/H1**
		**17: H2 (height of the dicrotic wave)**	**20: RI: H3/H1**
		**18: H3 (height of the diastolic peak)**	
	**Slope**	**21: RS (H1/T1)**	**22: FS (H1/(T–T1))**
		**23–44: Std of 1–22**.	**45–66: Rmssd of 1–22**.
	**Other features**	**67: *TW_a_*: area of tidal wave (TW)**	**69: *TW_vt_*: product of *TW_v_* and *TW_t_***
		**68: *TW_P_*: power of TW**	**70: *TW_st_*: stress index**
		**71–74: Std of 67–70**.	**75–78: Rmssd of 67–70**.
		**79: *TP_car_*: total power of cardiac component**	**80: *TP_res_*: total power of respiratory component**
**VPG and APG**	**Height**	**81: Hw (height of w point in VPG)**	**85: He (height of e point in APG)**
		**82: Hz (height of z point in VPG)**	**86: Hb/Ha**
		**83: Ha (height of a point in APG)**	**87: He/Ha**
		**84: Hb (height of b point in APG)**	**88: (Hb–He)/Ha**
		**89–96: Std of 81–88**.	**97–104: Rmssd of 81–88**.
**PRV**		**105: LF (low frequency power of PRV)**	**108: nLF (LF/(LF+HF))**
		**106: HF (high frequency power of PRV)**	**109: LF/HF**
		**107: TP (total power of PRV)**	**110: SampEn: sample entropy of pulse rate**

*VPG, first derivative or velocity of PPG; APG, second derivative or acceleration of PPG; PRV, pulse rate variability; Std, standard deviations; Rmssd, root mean square of the successive difference; PW, pulse width corresponding to the midpoint of the rising phase; PPT, peak to peak interval between the systolic and diastolic peaks; IPA, inflection point area ratio; TW, tidal wave; RS, rising slope of PPG; FS, falling slope of PPG*.

##### Features of PPG

For each cardiac cycle, 22 basic morphological parameters of PPG were acquired (Figure 1B, Table 1). We calculated their mean value, standard deviation (Std), which represents the overall variation, and root mean square of the successive difference (Rmssd) that represents local variation during 5 min of data acquisition.

In addition to the morphological features, the characteristics of the tidal wave (TW, Figure 1C) were also extracted. TW has a frequency ranging from 15 to 35 Hz and is often used as an index to indicate the hardening of blood vessels (Kageyama et al., 2007). Four basic features were calculated based on the TW: area of TW (*TW*_*a*_), power of TW (*TW*_*p*_), product of amplitude (*TW*_*v*_), time span (*TW*_*t*_) of TW (*TW*_*vt*_), and stress index (*TW*_*SI*_). We also extracted the mean value, Std, and Rmssd of the features over 5 mins of data acquisition (Figure 1C, Table 1). *TW*_*P*_ and *TW*_*SI*_ were defined and calculated as Equations 2.2 and 2.3:


(2.2) $$\begin{array}{r} TW_{P}\left(i\right)=\;\overset{N_{i}}{\underset{t=S_{i}}{\sum }}\overset{35\;Hz}{\underset{f=15\;Hz}{\sum }}MWT\left(PPG\right) \end{array}$$

(2.3) $$\begin{array}{r} TW_{SI}\;\left(i\right)=TW_{P}/\left[TW_{v}\left(i\right)*TW_{t}\;\left(i\right)\right] \end{array}$$

where MWT refers to Morlet-wavelet transform with the mother wavelet “cmor3-1.”

The wavelet analysis of the PPG signal identified two strong components that reflected cardiac and respiratory modulation of PPG (Dehkordi et al., 2018). A review of observational studies showed that the cardiac and frequency bands for children and young adults range from 0.50 to 3 Hz (30–180 beats/min) and 0.14 to 0.9 Hz (8–54 breaths/min), respectively, whereas in adults, they are more restricted (Fleming et al., 2011). Combined with the data of our experiment, we set the two ranges as 0.75 to 2.5 Hz (45–150 beats/min) for the cardiac component *(TP*_car_, as shown in Equation 2.4) and 0.1 to 0.6 Hz (6–36 breaths/min) for the respiratory component (*TP*_res_, as shown in Equation 2.5):


(2.4) $$\begin{array}{r} TP_{car}=\overset{300s}{\underset{t=0}{\sum }}\overset{2.5\;Hz}{\underset{f=0.75\;Hz}{\sum }}MWT\;\left(PPG\right) \end{array}$$

(2.5) $$\begin{array}{r} TP_{res}=\overset{300s}{\underset{t=0}{\sum }}\overset{0.6Hz}{\underset{f=0.1Hz}{\sum }}MWT\left(PPG\right) \end{array}$$

##### Features of APG and VPG

Studies on the derivatives of PPG (VPG and APG) have been conducted since the 1990s, and their amplitudes have been found to closely relate to vascular status (Takazawa et al., 1998). However, in studies that use PPG for physiological or pathological classification, only the features of PPG are used, and those of PPG derivatives are not (Pelaez et al., 2019; Seok et al., 2019). Because the vascular state is strongly associated with sympathetic nerves, we speculated that the use of the features of PPG derivatives would benefit sympathetic assessment. Eight amplitude features of VPG and APG, as well as their Std and Rmssd over 5 min of data acquisition, were extracted.

##### PR-Related Features

Suhrbier et al. (2006) recommended w–w interval (interval between the adjacent w points of the VPG) as the optimal surrogate for R–R interval when calculating PR and PRV. The calculation of PRV was the same as that of HRV.

Sample entropy (SampEn), proposed by Richman and Moorman (2000), is a nonlinear method used to evaluate the complexity of physiological time series:


(2.6) $$\begin{array}{r} SampEn=-\ln\left[\frac{B^{m+1}\left(r\right)}{B^{m}\left(r\right)}\right] \end{array}$$

where m is the pattern length and r is the similarity criterion. *B*^*m*^(*r*) is the number of two sets of simultaneous data points of length m that have a distance < r. SampEn of PR was obtained using *m* = 2 and *r* = 0.2^*^std.

### Feature Selection Using RF

Random forest is one of the most widely used machine learning algorithms and is highly efficient at solving classification problems without scaling the data or repeatedly adjusting the parameters (Breiman, 2001). Based on this, Genuer et al. (2010) proposed an interpretable RF model which is particularly useful when the sample size is small and the number of features is relatively large. Two types of feature sets can be acquired using this method: interpretation and classification features. The former contains all the important features that can help determine the underlying physiological mechanism, and the latter removes redundant interpretation features to include the least number of features that still enable good classification performance. The detailed process is described below (Genuer et al., 2010), and the related parameters include the number of trees in the forest (n_estimators), the maximum number of features of decision trees (max_features), and the setup of random seeds during program running (random_state).

The first step is feature ranking. The features are ranked by sorting the feature importance averaged over the 50 runs in descending order (n_estimators = 2,000, max_features = 0.3, random_state = 0–49).

The second step is feature elimination. The Std is calculated for the sorted feature importance in step one. Classification and Regression Tree (CART) model fitting is implemented on the Std results to obtain the threshold, which is defined as the minimum prediction value. Features with importance below this threshold are removed.

Step three is the selection procedure for the interpretation features. A series of models are built starting with the most important feature, and features are added to the model one at a time until all important features have been included. Features of the model that lead to the smallest out-of-bag (OOB) error are the interpretation features (n_estimators = 500, max_features = sqrt).

Step four is the selection procedure for the classification features. This procedure eliminates redundant interpretation features. Using sequential feature introduction, a variable is added only if the error gain exceeds a threshold (Equation 2.7). The threshold is set to the mean of the absolute values of the first-order differentiated OOB errors between the first model (after interpretation feature selection) and the model including all features after the elimination procedure (n_estimators = 500, max_features = sqrt, random_state = 0):


(2.7) $$\begin{array}{r} threshold=\frac{1}{p_{elim}-p_{interp}}\overset{pe\lim-1}{\underset{j=p_{interp}}{\sum }}|oob\left(j+1\right)-oob\left(j\right)| \end{array}$$

where *p*_*elim*_ is the number of features after feature elimination, and *p*_*interp*_ is the number of interpretation features.

According to the above method, we built classification models for the following four classification problems: BSL&SDB, BSL&CPT, BSL&MAT, and BSL&SDB&CPT&MAT. Validation of the classification models was carried out using 10-fold cross-validation. The number of trees in the forest (n_estimators) and the maximum number of features of decision trees (max_features) were determined using the grid search method, where the options for n_estimators ranged from 40 to 540 with a step size of 50, and the options for max_features were 0.3, “sqrt,” and 0.7. Because different random states lead to slightly different results, we implemented the above procedures 30 times in different random states (random state = 0–29) and calculated the average accuracy (AC). Python 3.8.2 (Python Software Foundation, Netherlands) was used for feature selection and model building.

### Using Sensitive PPG Features to Fit the Improved HRV Parameters Using Stepwise Regression

A growing body of research has suggested that the traditional HRV parameters are highly questionable (Billman, 2013). Therefore, we verified the feasibility of fitting the improved HRV parameters using the selected PPG features, which were the classification features of BSL&SDB&CPT&MAT. Using stepwise regression, we automatically selected the most important variables to establish the prediction model of the regression analysis. Variables were introduced one by one on the condition that the partial regression square sum was significant. The previously introduced variables were tested one by one, and the non-significant variables were removed. This process was repeated until no significant variables were selected or removed from the regression equation.

### Statistical Analysis

Differences between BSL and SDB, CPT, and MAT were compared using paired *t*-tests in the 28 subjects who had data of all four states (SPSS Statistics 22; IBM, USA). Statistical significance was represented as *p* < 0.05 and *p* < 0.01. Data are presented as means ± mean square error (MSE).

## Results

**Figure 2:**
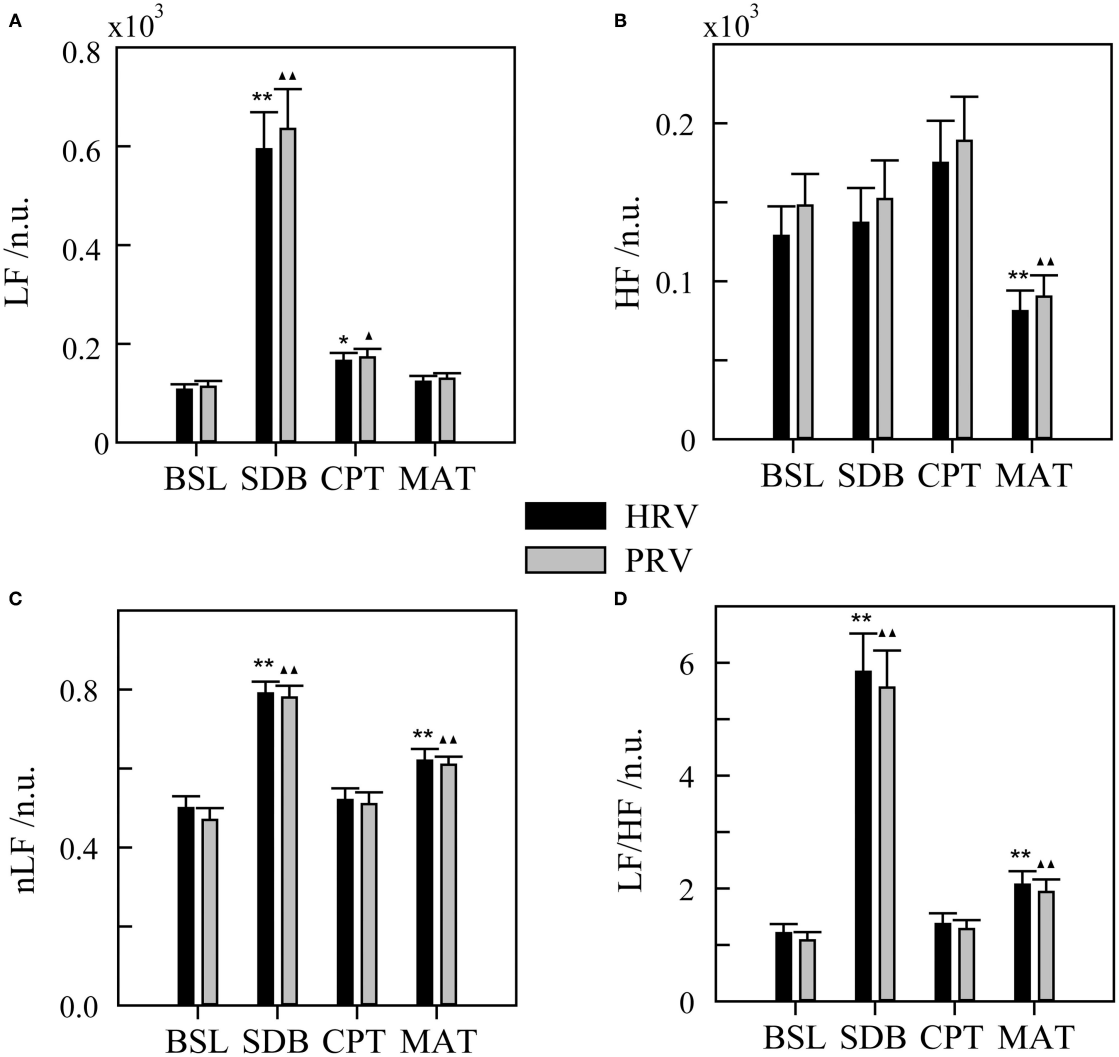
Heart rate variability (HRV) and pulse rate variability (PRV) for different ANS activation patterns. **(A)** LF, low-frequency power. **(B)** HF, high-frequency power. **(C)** nLF, normalized low-frequency power. **(D)** LF/HF, ratio of LF to HF. BSL, baseline state; SDB, slow deep breathing; CPT, cold pressor test; MAT, mental arithmetic test. The significance of the differences in HRV parameters between each experimental state and BSL state is denoted by * for *p* < 0.05 and ** for *p* < 0.01. ^▴^ and ^▴▴^ indicate *p* < 0.05 and *p* < 0.01, respectively, for the corresponding comparisons of the PRV parameters. The physiological mechanism of HRV parameters is considered as follows: LF mainly contains information on sympathetic activity, HF reflects parasympathetic activity, nLF is a relatively pure measure of sympathetic activity, and LF/HF reflects sympathovagal balance.

First, the results of HRV and PRV parameters under different ANS activation patterns were shown in Figure 2. The HRV method showed that LF, nLF, and LF/HF increased during SDB compared with that during BSL, which suggested significant sympathetic activation. And during MAT, HF decreased, whereas nLF and LF/HF increased compared with that during BSL, which suggested an increase of sympathovagal balance due to the decrease of parasympathetic activation. These results did not characterize the real changes in the ANS in the corresponding activation experiments (compared with that in the BSL states). The variation trends of the PRV parameters were consistent with those of the HRV parameters in different experiments. Meanwhile, there were also several differences. Specifically, the LF and HF of PRV were larger than those of HRV. For LF, this trend was most evident for SDB, whereas for HF, this was evident across all four experiments. Similarly, nLF and LF/HF of PRV were slightly smaller than those of HRV.

**Figure 3:**
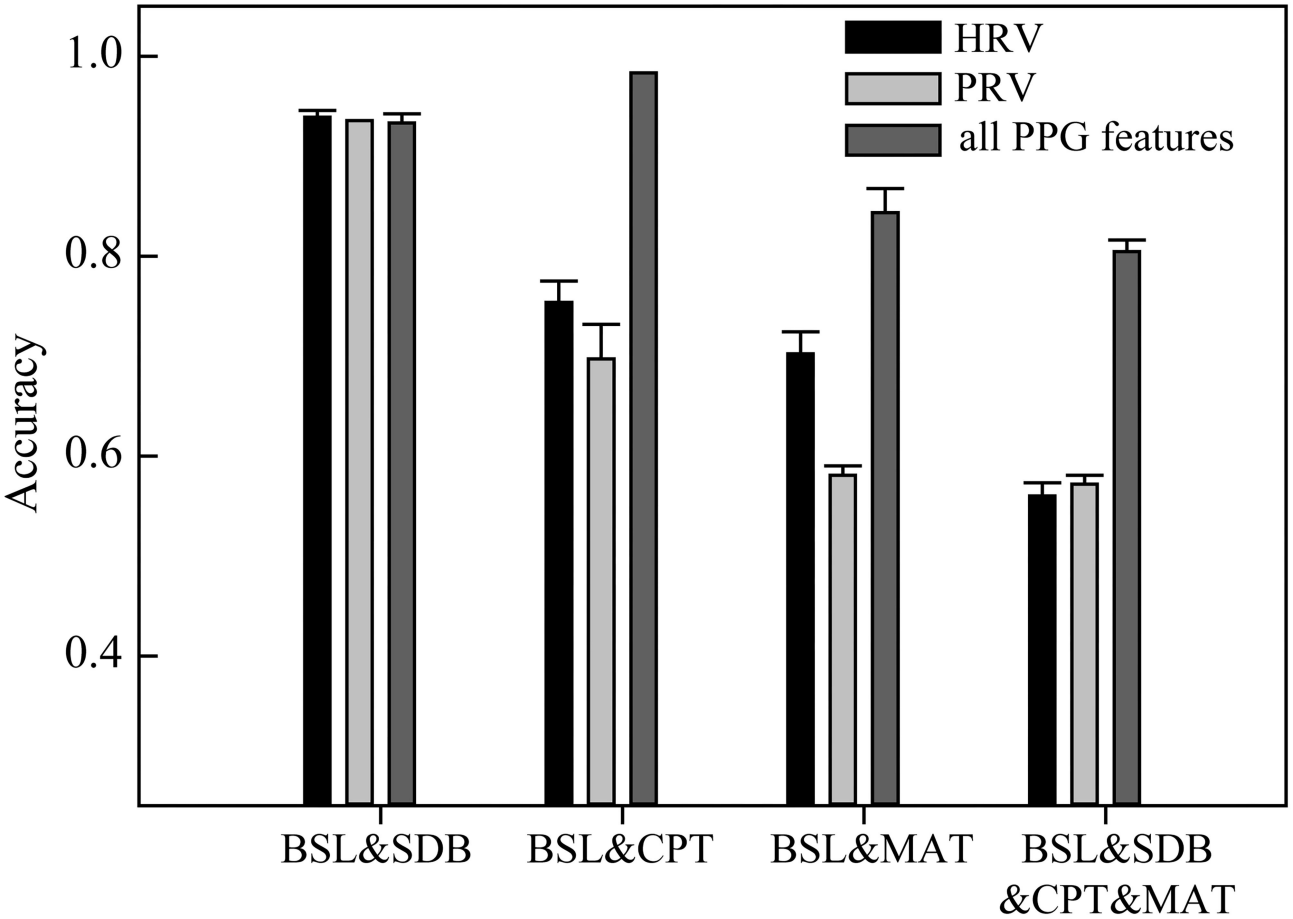
The classification accuracies of the models based on heart rate variability (HRV), pulse rate variability (PRV), and all PPG-based features (mean accuracy of 30 runs at different random states).

Heart rate variability, PRV, and all PPG-based features were used for the classification (Figure 3). HRV performed well for classifying BSL&SDB (accuracy > 0.93); however, it was not ideal for classifying BSL&CPT, BSL&MAT, or the four ANS activation patterns (BSL&SDB&CPT&MAT), as indicated by the classification accuracies of 0.75, 0.70, and 0.56, respectively. No improvement in classification performance was achieved when using PRV compared with that using HRV, especially for BSL&CPT and BSL&MAT, which showed significant decreases in accuracy. However, using all PPG-based features improved classification accuracies for BSL&CPT, BSL&MAT, and all the four ANS activation patterns to 0.98, 0.84, and 0.80, respectively, although accuracy remained unchanged for BSL&SDB.

**Figure 4:**
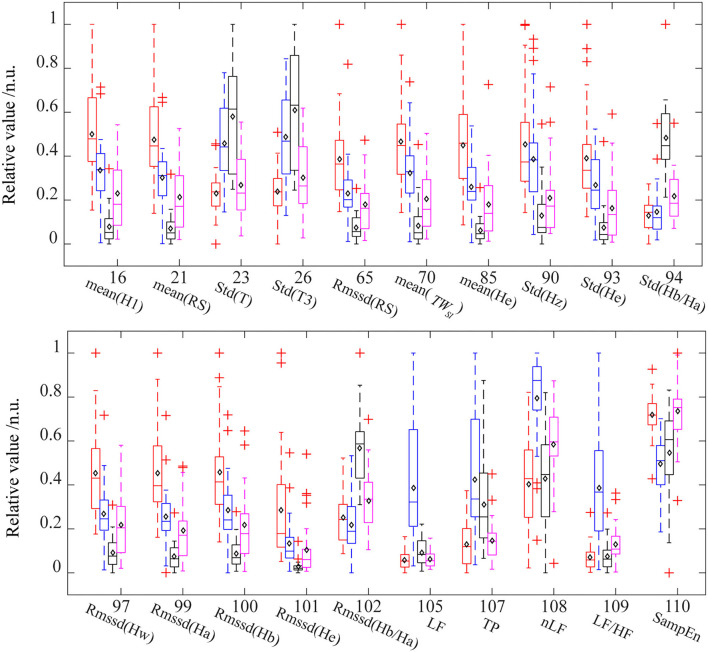
The distribution of interpretation features in different ANS states. For each feature, the data were normalized between the four states. Red (baseline state, BSL), blue (slow deep breathing, SDB), black (cold pressor test, CPT), and pink (mental arithmetic test, MAT). The diamond mark represents the mean value. Std, standard deviations; Rmssd, root mean square of the successive difference; H1, height of the systolic peak; RS, rising slope of PPG; T, time span of the pulse; T3, diastolic time of the pulse; *TW*_*st*_, stress index; Hw, height of w point in VPG; Hz, height of z point in VPG; Ha, height of a point in APG; Hb, height of b point in APG; He, height of e point in APG; SampEn, sample entropy of pulse rate; LF, low-frequency power of PRV; TP, total power of PRV. nLF, normalized low-frequency power; LF/HF, ratio of LF to HF.

Table 2 lists the interpretations and classification features for the four classification models that used all the PPG-based features. Interpretation features were a set of features with the highest single-feature contribution rate. Overall, there were 20 interpretation features, which were listed in Table 2, and their variations among the four ANS states were depicted in Figure 4.

**Table 2:** Classification models based on all PPG features.

**Classification problem**	**Accuracy**			**Interpretation features**	**Classification features**
	**Mean**	**Max**	**Min**		
BSLandSDB	0.93	0.95	0.90	110(SampEn), 105(LF), 109(LF/HF), 108(nLF), 107(TP), 23[Std(T)], 26[Std(T3)], 99[Rmssd(Ha)], 65[Rmssd(Rs)], 97[Rmssd(Hw)]	110, 105, 109, 23, 26
BSLandCPT	0.98	0.98	0.98	99[Rmssd(Ha)]	99
BSLandMAT	0.84	0.87	0.78	85[Mean(He)], 101[Rmssd(He)], 90[Std(Hz)], 108(nLF), 109(LF/HF), 99[Rmssd(Ha)], 100[Rmssd(Hb)], 70[Mean(*TW_st_*)], 93[Std(He)], 16[Mean(H1)]	85, 90, 108, 99
BSLandSDBandCPTandMAT	0.80	0.84	0.78	105(LF), 94[Std(Hb/Ha)], 110(SampEn), 109(LF/HF), 108(nLF), 99[Rmssd(Ha)], 107(TP), 85[Mean(He)], 102[Rmssd(Hb/Ha)], 21[Mean(Rs)]	105, 94,109, 99, 102, 23

*The interpretation and classification features corresponding to the highest classification accuracy in 30 runs are listed. For those that had more than 10 interpretation features, only the top 10 are listed. Features are presented in descending order of feature importance*.

*BSL, baseline state; SDB, slow deep breathing; CPT, cold pressor test; MAT, mental arithmetic test; Mean, mean accuracy of 30 runs at different random states; Max, maximum accuracy; Min, minimum accuracy; Std, standard deviations; Rmssd, root mean square of the successive difference; SampEn, sample entropy of pulse rate; LF, low-frequency power of PRV; TP, total power of PRV; nLF, normalized low-frequency power; LF/HF, ratio of LF to HF; T, time span of the pulse; T3, diastolic time of the pulse; TW_st_, stress index; H1, height of the systolic peak; RS, rising slope of PPG; Hw, height of w point in VPG (first derivative or velocity of PPG); Hz, height of z point in VPG; Ha, height of a point in APG (second derivative or acceleration of PPG); Hb, height of b point in APG; He, height of e point in APG*.

Table 2 shows that the important interpretation features for BSL&SDB (parasympathetic activation) were SampEn, LF, LF/HF, nLF, TP, and Std(T), all of which were associated with PR. For the classification of BSL&CPT (peripheral sympathetic activation), although most features differed significantly (Figure 4), Rmssd(Ha) was the only interpretation and classification feature, which achieved the optimal accuracy without the cooperation of other features. For the classification of BSL&MAT (sympathetic activation), the important interpretation features were the three amplitude features of the PPG derivatives [Mean(He), Rmssd(He), and Std(Hz)], followed by the two PRV features (nLF and LF/HF). Besides the features mentioned above, the interpretation features of BSL&SDB&CPT&MAT also included two features of Hb/Ha [Std(Hb/Ha) and Rmssd(Hb/Ha)], which were significantly increased in CPT than in the other three states, and indicated that Hb/Ha was a favorable feature of peripheral sympathetic activation.

The classification features of BSL&SDB&CPT&MAT comprised the best feature collection capable of distinguishing the four different ANS activation patterns and were used to fit the improved HRV parameters (Table 3). The fitting features for LFru were LF, Std(He), and Std(T); and those for HFr were LF, LF/HF, and Std(T). The *p*-value and *F*-value results showed that the two regression models were both acceptable.

**Table 3:** Using sensitive PPG features to fit the improved HRV parameters.

		**LFru**	**HFr**
Fitting parameters	Intercept	−250.5855	25.7447
	LF	−0.2786	1.2110
	Std(Hb/Ha)	2.5954e+03	0
	LF/HF	0	−28.7423
	Rmssd(Ha)	0	0
	Rmssd(Hb/Ha)	0	0
	Std(T)	6.0328	1.1627
Model evaluation	*F*-value	47.6866	452.363
	*p*-value	2.9264e−20	1.5981e−64

*At p < 0.05, the standard F-value was F_(122,3)_ = 2.6789*.

*LF, low-frequency power of PRV; LF, high-frequency power of PRV; LF/HF, ratio of LF to HF; T, time span of the pulse; Ha, height of a point in APG (second derivative or acceleration of PPG); Hb, height of b point in APG*.

**Figure 5:**
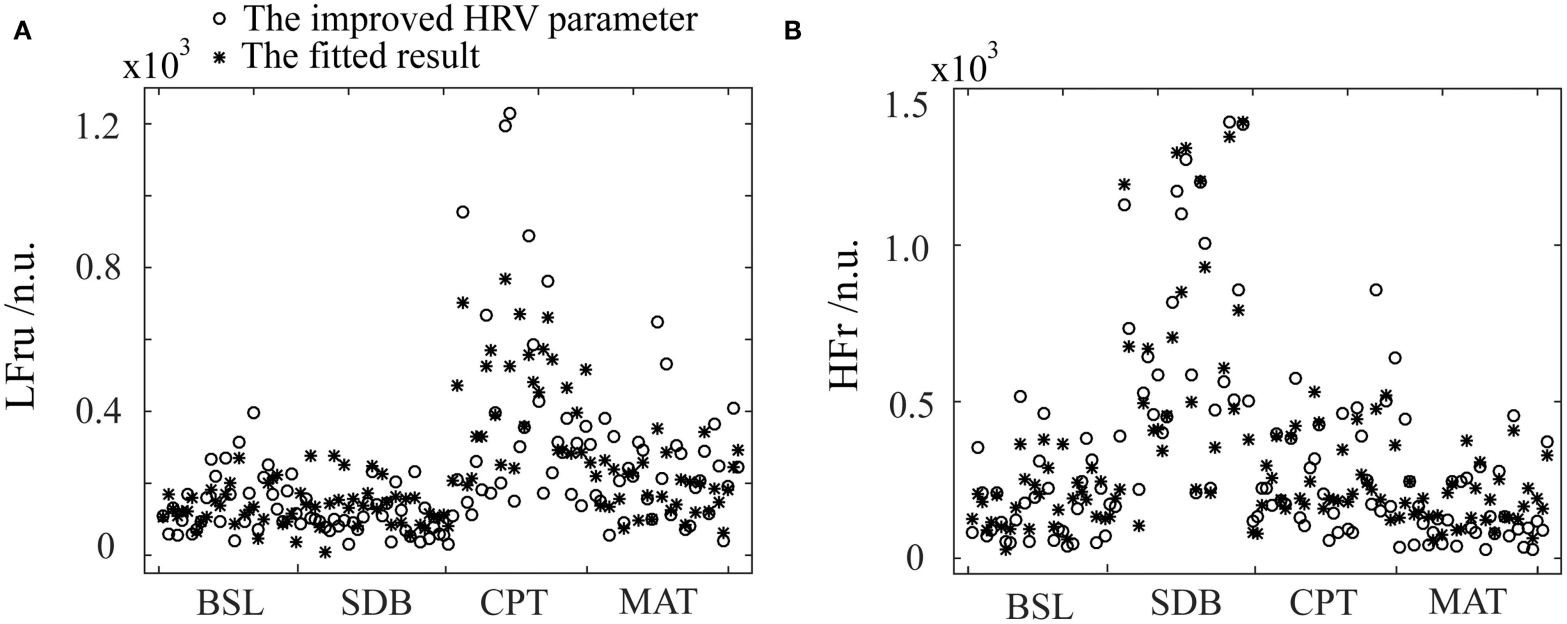
The fitting results of the improved HRV parameters using the PPG features and stepwise regression. **(A)** LFru, low-frequency power of the improved HRV. **(B)** HFr, high-frequency power of the improved HRV. BSL, baseline state; SDB, slow deep breathing; CPT, cold pressor test; MAT, mental arithmetic test. The physiological mechanism of the improved HRV parameters are considered as: LFru mainly contains information on sympathetic activity, HFr reflects parasympathetic activity.

The specific fitting results were shown in Figure 5. We observed that the data of the same parameter (i.e., LFru or HFr) varied considerably depending on the experimental settings. In detail, LFru of the CPT and MAT was significantly higher than that of BSL and the SDB, and the HFr of the SDB was significantly higher than that of the other three experiments. It could also be seen that the fitting results obtained by using the ANS-sensitive PPG-based features could well reproduce the variation trend of the two parameters in different experiments. The fitting of the LFru was relatively inferior to that of the HFr, which was not satisfactory especially for the LFru of the CPT.

**Figure 6:**
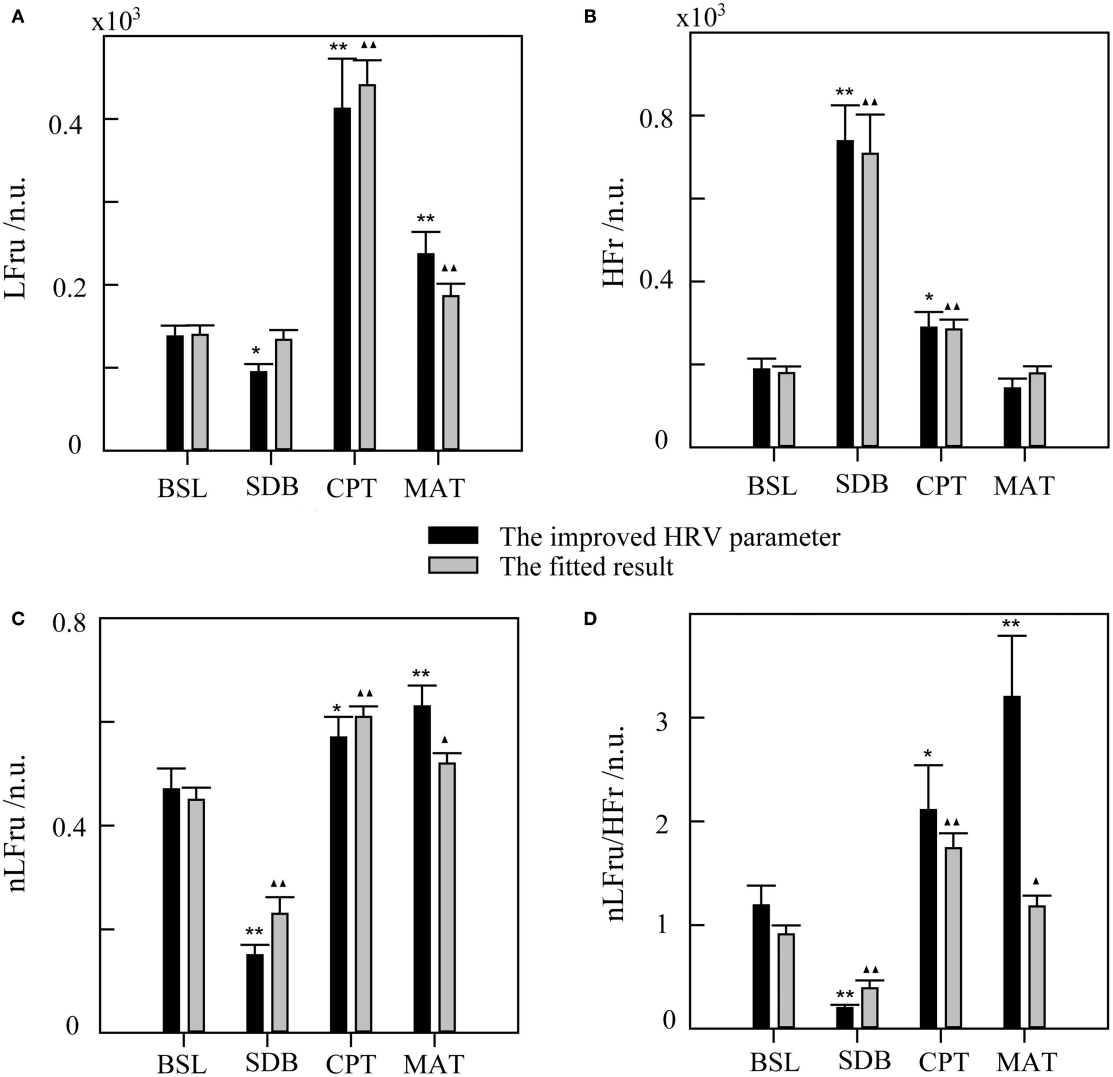
The statistical results of slow deep breathing (SDB), the cold pressor test (CPT), and the mental arithmetic test (MAT) compared with the baseline state (BSL) for the improved HRV parameters and fitting data. **(A)** LFru, low-frequency power of the improved HRV. **(B)** HFr, high-frequency power of the improved HRV. **(C)** nLFru, normalized low-frequency power. **(D)** LFru/HFr, the ratio of LFru to HFr. The significance of the differences in the improved HRV parameters between each experimental state and BSL state is denoted by * for *p* < 0.05 and ** for *p* < 0.01. ▴ and ▴▴ indicate *p* < 0.05 and *p* < 0.01, respectively, for the corresponding comparisons of the fitted result. The physiological mechanism of the improved HRV parameters are considered as follows: LFru mainly contains information on sympathetic activity, HFr reflects parasympathetic activity, nLFru is a relatively pure measure of sympathetic activity, and LFru/HFr reflects sympathovagal balance.

The statistical results of the SDB, CPT, and MAT compared with those of BSL, based on the improved HRV parameters and fitting data, were shown in Figure 6. The improved HRV method showed that HFr increased, whereas LF, nLF, and LF/HF decreased during SDB compared with that during BSL, which indicated significant parasympathetic activation. In contrast, LF, nLF, and LF/HF increased significantly during MAT compared with that during BSL, which indicated sympathetic activation. These results characterized the real changes in the ANS in the corresponding activation experiments (compared with that in the BSL). For LFru, HFr, and their corresponding fitting data, although there were some differences in the mean values, little difference was found for the variation trends. Relatively more differences were found in the derived parameters, nLFru and LFru/HFr, and their fitting data. Despite these differences, the fitting results could still highlight the corresponding changes of ANS in each experiment.

## Discussion

In this study, we explored the utility of PPG signals for ANS assessment. The classification of different ANS activation patterns was achieved using PPG-based features, and we obtained ANS-related PPG features. Then, these features were used to fit the improved HRV parameters, and a model to estimate ANS activation was developed.

Heart rate variability has been widely used to evaluate ANS activity. However, it was found that LF has a weak relationship with sympathetic activity because it contains multiple regulatory information, which includes the sympathetic system, parasympathetic system, and some undefined factors (Randall et al., 1991; Houle and Billman, 1999; Goldstein et al., 2011). In contrast, HF is strongly influenced by respiratory rate and may exceed the defined frequency band (0.15–0.4 Hz) in many cases (Berntson et al., 1993). These flaws were clearly exposed, as shown in Figure 2. The LF of the SDB group increased significantly because the parasympathetic regulation-related spectrum shifted to the LF band with decreased respiratory rate. In the MAT group, HF significantly decreased and the changes in LF were slight, which may have been due to the high proportion of parasympathetic regulation in LF.

When considering using PPG for ANS evaluation, most studies have focused on evaluating the possibility of replacing HRV with PRV (Schafer and Vagedes, 2013). The basic conclusion is that time and frequency parameters of PRV and HRV are interchangeable only in the supine state but not in other states, such as head-up tilt, standing, cold stimulation, mental stress, and during exercise (Giardino et al., 2002; Charlot et al., 2009; Gil et al., 2010; Iozzia et al., 2016; Pernice et al., 2019; Mejía-Mejía et al., 2020). Although studies vary considerably because of diverse experimental settings and/or analysis methods, a unifying result is that the spectrum power of PRV (especially the HF band) is always larger than that of HRV (Schafer and Vagedes, 2013), which was also evident in our study (Figure 2). Given the limitations of HRV, other approaches to evaluate ANS activation are needed.

Photoplethysmography measures the changes in blood volume in the fingertips and is related to cardiac and vascular performance, both of which are mainly subjected to the regulation of the ANS (Avolio, 2002). Therefore, other features of PPG besides PRV can also provide information about ANS activity from more perspectives. Results showed a significant improvement in the classification accuracy after using more PPG-based features compared with that using PRV (Figure 3). On a complete PPG waveform, the horizontal axis corresponds to the entire cardiac cycle (T) and its internal details, such as systole (T2) and diastole (T3). The longitudinal axis (or amplitude) of the PPG waveform represents the change in blood volume, which is related to stroke volume and the state of the peripheral blood vessels. In particular, the amplitude-related features of VPG and APG are more sensitive to vascular lesions (Bortolotto et al., 2000; Baek et al., 2007) and vascular resistance (Takazawa et al., 1998).

Slow deep breathing is often used as a stress reduction technique in previous studies (Gilbert, 2003; Busch et al., 2012). The existing researches indicate that slow breathing enhances activation of the parasympathetic nervous system (Pal et al., 2004), which has far more effects on HR than on the vascular states. As a result, it leads to a decrease in average HR (Jerath et al., 2006) and an increase in HRV (Jan et al., 2019), which are also evident in our results. Figure 4 showed that in the SDB compared with that in the BSL, the pulse rate-related features [Std(T), Std(T3), LF, TP, nLF, LF/HF, and SampEn] changed more significantly than the other features. And the interpretation features of BSL&SDB were the time domain [Std(T) and Std(T3)], the frequency domain (LF, LF/HF, nLF, and TP), and the nonlinear domain (SampEn) features of pulse rate (Table 2). Therefore, parasympathetic activation induced by SDB primarily changed the horizontal axis of the PPG waveform (or cardiac cycle), especially the diastole [Std(T3)].

For the CPT, the intense cold stimulation activated the baroreflex modulation (Cui et al., 2002; Incognito et al., 2019) and high-threshold nociceptive fibers (Kregel et al., 1992), which might cause a more marked increase in muscle sympathetic nerve activity (MSNA) if compared with SDB and MAT. As a result, the most obvious changes caused by CPT were concentrated on the amplitude-related features [mean(H1), mean(Rs), Rmssd(Rs), mean(He), Std(Hz), Std(He), Std(Ha/Ha), Rmssd(Hw), Rmssd(Ha), Rmssd(Hb), Rmssd(He), and Rmssd(Hb/Ha)]. Among them, only Rmssd(Ha) was selected as an interpretation feature for BSL&CPT (Table 2). Ha is the amplitude of the first peak of APG and corresponds to the position where the PPG signal has the highest acceleration during the rising stage.

Although MAT mainly activates cardiac sympathetic nerves, it also influences peripheral sympathetic activity (Anderson et al., 1987). Our data (Figure 4) indicated that several amplitude features exhibited greater changes than did PRV features. Of the important interpretation features (Table 2), nLF and LF/HF were ranked fourth and fifth, whereas almost all other features were amplitude features [mean(He), Rmssd(He), and Std(Hz)]. This might be partly due to the high sensitivity of the fingers to MSNA and partly due to the limited accuracy of PRV parameters and their big individual differences. Mejía-Mejía et al. (2020) also suggested that PPG in peripheral sites such as the finger and the toe may have different information not available in HRV. From the feature importance of BSL&MAT, some amplitude features [mean(He), Rmssd(He), and Std(Hz)] of VPG&APG may be superior markers of sympathetic activation than are nLF and LF/HF.

The classification features of BSL&SDB&CPT&MAT consisted of sensitive features of both parasympathetic and sympathetic activities. The fitting of the improved HRV demonstrated the feasibility of ANS evaluation using these PPG features because it reflected ANS activity more accurately than did traditional HRV. However, we did not use additional datasets to test the regression models, which might be one of the limitations. On the contrary, our data included four different ANS activation states, each with distinct changes in LFru (or HFr, Figure 5). In this context, the two models still showed good performance with *p*-values much < 0.01 (Table 3), which illustrated the validity of the models from another perspective. In addition, we only included data of 32 healthy young men, which would limit the generalizability of our conclusions. In the future, more data from a wider population are needed to establish more accurate and practical prediction models.

## Conclusion

Photoplethysmography offers promising applications in both clinical and home monitoring. Since PRV has been proved with limited accuracy in characterizing ANS activities, it is necessary to consider the use of PPG in ANS from another perspective. The results of the classification models showed that there were also many other PPG features that were sensitive to ANS activities. Finally, by combining such features in regression models to fit the improved HRV parameters, it might be possible to enhance the potential of PPG for ANS assessment.

## Data Availability Statement

The datasets generated for this study are available on request to the corresponding author.

## Ethics Statement

The studies involving human participants were reviewed and approved by Xi'an Jiaotong University Ethics Committee. The patients/participants provided their written informed consent to participate in this study.

## Author Contributions

BL contributed to the study design, data collection, analysis and interpretation of the data, and writing of the report. ZZ contributed to the data collection and analysis. XD, XW, and LX contributed to the data collection and interpretation. WX contributed to the critical revision of the manuscript. JZ contributed to the study design and critical revision of the manuscript. All authors read and approved the final manuscript to be submitted.

## Funding

This work was supported by the National Natural Science Foundation of China (Grant 11872297).

## Conflict of Interest

The authors declare that the research was conducted in the absence of any commercial or financial relationships that could be construed as a potential conflict of interest.

## Publisher's Note

All claims expressed in this article are solely those of the authors and do not necessarily represent those of their affiliated organizations, or those of the publisher, the editors and the reviewers. Any product that may be evaluated in this article, or claim that may be made by its manufacturer, is not guaranteed or endorsed by the publisher.
